# Prognostic Value of the B12/CRP Index in Older Systemically Treatable Cancer Patients

**DOI:** 10.3390/cancers14010169

**Published:** 2021-12-30

**Authors:** Coline Montegut, Florian Correard, Emilie Nouguerède, Dominique Rey, Thomas Chevalier, Marie Meurer, Jean-Laurent Deville, Marjorie Baciuchka, Vincent Pradel, Laurent Greillier, Patrick Villani, Anne-Laure Couderc

**Affiliations:** 1 Internal Medicine, Geriatrics and Therapeutic Unit, Assistance Publique des Hôpitaux de Marseille (AP-HM), 13009 Marseille, France; Coline.MONTEGUT@ap-hm.fr (C.M.); emilie.nouguerede@ap-hm.fr (E.N.); dominique.rey@ap-hm.fr (D.R.); patrick.villani@ap-hm.fr (P.V.); 2 Coordination Unit for Geriatric Oncology (UCOG), PACA West, 13009 Marseille, France; 3 Pharmacy Department, Assistance Publique-Hôpitaux de Marseille (AP-HM), 13005 Marseille, France; florian.correard@ap-hm.fr; 4 Oncology Unit, Assistance Publique-Hôpitaux de Marseille (AP-HM), 13005 Marseille, France; thomas.chevalier@p-hm.fr (T.C.); marie.meurer@ap-hm.fr (M.M.); Jean-laurent.DEVILLE@ap-hm.fr (J.-L.D.); 5 Multidisciplinary Oncology and Therapeutic Innovations Department, Assistance Publique-Hôpitaux de Marseille (AP-HM), 13015 Marseille, France; marjorie.baciuchka@ap-hm.fr (M.B.); laurent.greillier@ap-hm.fr (L.G.); 6 Public Health Department, Assistance Publique-Hôpitaux de Marseille (AP-HM), 13005 Marseille, France; vincent.pradel@ap-hm.fr; 7 Marseille Cancer Research Center (CRCM), National Institute of Health and Medical Research (INSERM), National Center for Scientific Research (CNRS), Aix-Marseille University, 13009 Marseille, France; 8 Anthropology Bio-Cultural, Law and Ethics (ADES), French Blood Agency (EFS), National Center for Scientific Research (CNRS), Aix-Marseille University, 13009 Marseille, France

**Keywords:** medical oncology, C-reactive protein, serum vitamin B12 level, frailty, older patients

## Abstract

**Simple Summary:**

Decisions on cancer treatment for older patients take into account not only comorbidities but also physical and cognitive resources. Comprehensive geriatric assessment (CGA) in older patients assesses geriatric frailties but does not include standardized biological tests. The B12/CRP index (BCI) was first intended as a prognosis tool to predict 90-day mortality after advanced cancer diagnosis. This study shows the prognostic link between BCI value and overall cancer survival time in older people, and the association between BCI value and geriatric frailty before cancer treatment in this population.

**Abstract:**

Background: While comprehensive geriatric assessment (CGA) in older patients treated for cancer assesses several related domains, it does not include standardized biological tests. The present study aimed to: (1) assess the prognosis value of the B12/CRP index (BCI) in a population of systemically treatable older patients with cancer and (2) analyze the association between BCI value and pre-existing geriatric frailty. Method: We conducted a retrospective observational study between January 2016 and June 2020 at Marseille University Hospital. All consecutive cancer patients aged 70 years and over before initiating systemic therapy were included. Results: Of the 863 patients included, 60.5% were men and 42.5% had metastatic stage cancer. Mean age was 81 years. The low-BCI group (≤10,000) had a significantly longer survival time than the mid-BCI (10,000 < BCI ≤ 40,000) and high-BCI (BCI > 40,000) groups (HR = 0.327, CI95% [0.26–0.42], *p*-value = 0.0001). Mid- and high-BCI (BCI > 40,000) values were associated with impaired functional status and malnutrition. Conclusion: A BCI > 10,000 would appear to be a good biological prognostic factor for poor survival times and pre-existing geriatric impairment in older cancer patients before they initiate systemic treatment.

## 1. Introduction

Patients >65 years of age account for nearly 50% and 60% of new cancer cases in the USA and Europe, respectively [1]. Oncologists and geriatricians have been working together in recent years to integrate Comprehensive Geriatric Assessment (CGA) into oncological practice for cancer patients. The International Society of Geriatric Oncology (SIOG) and the National Comprehensive Cancer Network (NCCN) currently recommend systematically performing a CGA in patients before initiating systemic cancer treatment [2,3]. In the American Society of Clinical Oncology (ASCO) guidelines, the expert panel recommends using validated tools which assess specific geriatric domains in order to predict shorter survival times in older cancer patients receiving treatment. However, recommendations for this population do not include using standardized biological analyses to estimate long-term prognosis [4]. 

Cancer-associated inflammation [5] adds to “inflamm-aging” [6,7] and may negatively impact survival. C-reactive protein (CRP) is synthesized by hepatocytes in response to pro-inflammatory cytokines such as interleukin 6, interleukin 1 and tumor necrosis factor-α. In the literature, CRP is one of several inflammatory biomarkers associated with frailty in older patients with cancer [8]. High CRP levels are associated with early death after diagnosis [9]. 

In addition, several studies have reported an association between either high serum vitamin B12 levels or hypercobalaminemia (HCbl) HCbl and poor prognosis in patients with solid tumors and hematological malignancies [10], particularly in older patients [11]. HCbl is related to an excess synthesis of transcobalamins by the tumor and/or an increase in haptocorrin secondary to hyperleukocytosis [12,13]. 

Some studies have shown the usefulness of measuring the vitamin B12/CRP index (B12 vitamin × CRP) (known as BCI) in the management of older cancer patients [10,14]. A BCI > 40,000 has been associated with poor survival time in persons with advanced cancer. In older patients, it is also associated with an increased risk of one- and three-month unplanned hospitalizations, as well as three-month mortality following cancer treatment initiation (irrespective of cancer type, stage and treatment) [15]. 

We performed an observational study to (1) assess the prognosis value of BCI in a population of systemically treatable older patients with cancer and (2) analyze the association between BCI value and pre-existing geriatric frailty.

## 2. Materials and Methods

### 2.1. Study Design and Participants

This retrospective observational study was conducted at Marseille University Hospital on all consecutive cancer patients aged 70 years and older referred to a geriatrician for a geriatric comprehensive assessment (CGA) before systemic treatment initiation, between January 2016 and June 2020. All the patients were registered at baseline in accordance with the French Database and Privacy Law (Commission Nationale de l’Informatique et Liberté CNIL registration number: 20-324).

### 2.2. Data Collection 

A geriatrician evaluated the different components of the CGA: functional status was assessed via the Activities of Daily Living (ADL) (impaired < 6/6) [16] and Instrumental Activities of Daily Living (IADL) (impaired < 4/4) scales [17]; Cognitive disorders and mood impairment were assessed by the Mini Mental State Examination (MMSE) (impaired MMSE < 24) [18], and short Geriatric Depression (impaired GDS ≤ 1/4) scales, respectively [19]; Body Mass Index (BMI), albumin levels, and the Mini Nutritional Assessment (MNA) scale [20] were used to determine nutritional status, as defined by the French Department of Health (malnutrition = BMI < 21 and/or albumin levels < 35 g/L and/or MNA < 17); Patients’ mobility was assessed using gait speed (impaired < 0.8 m/s) [21], the Timed ‘Up and Go’ Test (impaired TUG > 20 s) [22], the One Leg Balance Test (impaired OLBT < 5 s) [23], fall history in the previous three months [24,25,26], and handgrip strength (impaired < 27 kg for men; <16 kg for women) [27]). Polypharmacy and high (i.e., three or four) modified Cumulative Illness Rating Scale (CIRS) scores [28] were also collected. Patients were asked about their demographic characteristics and lifestyle (age, living place, and presence of a caregiver). 

Data on cancer site, stage and biological data were obtained from medical records. A biological assessment was performed at the same time as the geriatric evaluation. Renal function was assessed using the Cockcroft creatinine clearance formula (severe renal failure if clearance < 30 µmol/L), anemia was defined by a level of hemoglobin <12 g/dL for women and <13 g/dL for men, thrombopenia was defined by a platelet level <150 G/L and lymphopenia by a lymphocytes level <1.26 G/L. Finally, the BCI was calculated as the product of the vitamin B12 (pmol/L) and the C-reactive protein (mg/L) levels:

BCI=Vitamin B12 (pmol/L) × C−reactive protein (mg/L)

In line with the literature, we studied BCI levels according to three categories: low-BCI (i.e., ≤10,000), mid-BCI (>10,000 and ≤40,000), and high-BCI (>40,000) [10,14]. Vitamin B12 was considered normal between 145 and 569 pmol/L; CRP was considered normal under 5 mg/L.

### 2.3. Statistical Analysis

A descriptive analysis was performed to describe our population according to the BCI. Different categorical variables were expressed in terms of the number or the percentage of patients. The association between variables was assessed using either the χ² test or the Fisher’s exact test as appropriate. A logistic regression model was performed to evaluate the association of different geriatric characteristics impairment to each BCI group using odd ratios adjusted (aOR) for age, gender and cancer stage. An exploratory Cox proportional hazards regression analysis—adjusted for age, gender and cancer stage—was performed to estimate adjusted hazard ratios (aHR) with corresponding 95% CIs from the date of the CGA to the date of death or date of last known contact before the database lock date (15 June 2021). A multicollinearity analysis was undertaken to assess confounding factors. The Kaplan-Meier method was used to estimate survival curves and the log-rank test to compare survival curves. All statistical analyses were performed using SPSS software (version 17.0). Significance was considered for *p*-values < 0.05. 

## 3. Results

### 3.1. Population

**Figure 1:**
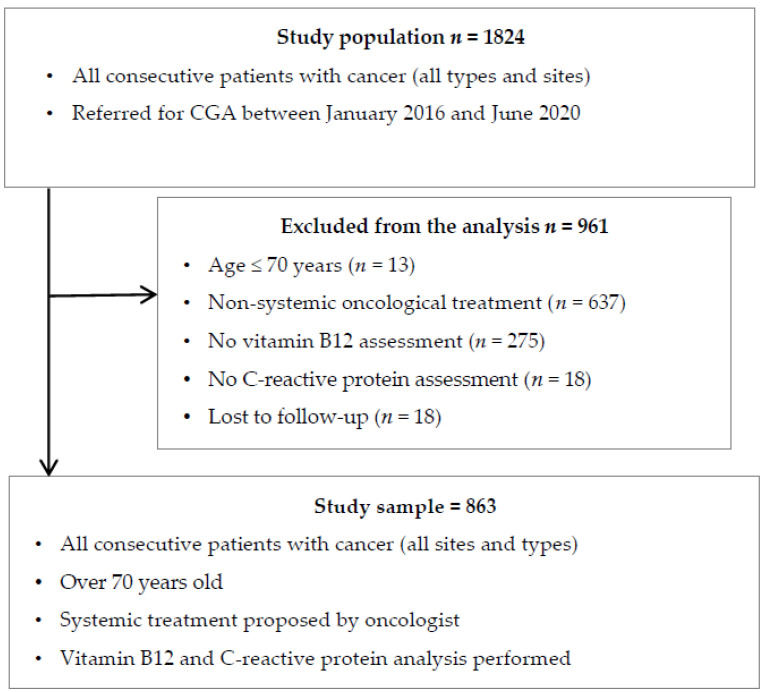
Flow chart.

A total of 1824 patients who had a CGA and did not receive vitamin B12 supplement in the weeks preceding CGA comprised our study population. Of these, we secondarily excluded 637 patients who were referred for surgery, radiotherapy only, or palliative care, 275 patients whose B12 vitamin blood level had not been assessed, 18 patients with no assessment of C-reactive protein blood levels, 13 patients aged under 70 years, and 18 patients lost to follow-up. The patients excluded from the analysis for lack of B12 or CRP dosage or lost to follow-up were younger, had less prostatic and gastrointestinal cancers, and less metastatic stages than the included population. They were comparable in terms of geriatric characteristics, but had less frequency of severe comorbidities (detailed in Table S1). The study sample therefore comprised 863 patients (Figure 1). 

The median follow-up time for our study was 31 months. Mean age of the study sample was 81 years (SD 5.9; range [70–100)), 60.5% were men, and 42.5% had metastatic disease stage (Table 1). Cancer sites are reported in Table 1, detailed treatment proposal is described in Table S2. The most frequent cancer types were prostate (21.3%), lung or thoracic (21.2%), gastrointestinal (15.2%), and breast (10.4%). 

Hypercobalaminemia was present in 14.8% of the sample, and half (54.5%) had high CRP (i.e., ≥5 mg/L). With regard to BCI, 76.1% of the sample were classified in the low-BCI group (i.e., BCI ≤ 10,000), 17% in the mid-BCI group (i.e., 10,000 < BCI ≤ 40,000), and 6.9% in the high-BCI group (i.e., BCI > 40,000). 

Almost two thirds of the sample (62.4%) had impaired autonomy (26% having either impaired ADL or IADL, with 36.4% having both impaired ADL and IADL). Similarly, 63.8% had cognitive impairment; and 46.3% had suspected mood impairment. Handgrip strength was impaired in 44.1% of the patients, 41.9% presented a Timed ‘Up and Go’ test over 20 s, 65.8% could not balance on one leg for at least 5 s, and 51.2% had a gait speed <0.8 m/s. One third (33.6%) of the study sample had malnutrition. With regard to hematology, 63.5% were anemic, 10.0% had thrombocytopenia, 39.0% lymphopenia and 12.4% severe renal deficiency (Table 1). 

### 3.2. BCI and Geriatric Frailty

Increased BCI was mainly associated with poor health status (Table 1). As expected, patients in the high-BCI group (i.e., >40,000) were mostly at a metastatic stage (62.1% of them had stage IV disease). Thoracic and gastrointestinal cancers were the most frequent in the high-BCI group. A high BCI value was also associated with impaired autonomy (71.2% had both impaired ADL and IADL), and mood impairments (79.6% and 61.8% had suspected cognitive or mood impairment, respectively), muscle strength loss (71.4% had impaired handgrip strength), and mobility impairment (69.6% could not perform the TUG test in under 20 s, 88.2% could not balance on one leg for 5 s or more, and 71.7% patients had a gait speed of <0.8 m/s). Malnutrition was also more frequent in these patients (81.4% were malnourished). Polypharmacy was also associated with higher BCI (77.4% of patients of the mid-BCI group and 74.6% of the high-BCI group were treated with five or more drugs), whereas the presence of at least one comorbidity wasn’t significantly associated to increased BCI levels (*p* = 0.156). Anemia was present in both the mid-BCI (10,000 < BCI ≤ 40,000) and high-BCI groups (77.6% and 88.1%, respectively) as were lymphopenia (46.5% and 55.9%, respectively) and severe renal deficiency (21.1% and 16.9% having a Cockcroft clearance <30 mL/min, respectively) (Table 1). 

To evaluate the usefulness of BCI as a prognostic factor for cancer-potentiated frailty, we performed a multinomial regression analysis with low BCI (i.e., BCI ≤ 10,000) as the reference. As impaired autonomy and/or malnutrition and/or impaired mobility were all statistically associated in our study, we used gender, age and cancer stage in three different models for autonomy, malnutrition and gait speed (which is a marker for mobility impairment). 

The results of the univariate comparative analysis were confirmed, with ADL and IADL impairment and malnutrition both strongly associated with mid and high BCI levels (Table 2 models A and B). Gait speed impairment was only associated with high BCI (Table 2 model C). The results in Table 2 also show that high BCI values were more strongly associated with loss of autonomy than were mid BCI values (aOR = 14.375, CI95% [5.80–35.66] vs. aOR = 4.069, CI95% [2.50–6.63], *p*-value = 0.0001). The same was observed for malnutrition (aOR = 14.348, CI95% [7.16–28.74] vs. aOR = 4.969, CI95% [3.30–7.37], *p*-value = 0.0001) (Table 2 model A and B). Mid BCI values were significantly associated with male gender and cancer stage (aOR = 1.950, IC95% [1.30–2.93], *p*-value = 0.001 and aOR = 2.452, IC95% [1.68–3.59], *p*-value = 0.0001, respectively). High BCI were also associated with male gender and cancer stage (aOR = 1.843, IC95% [1.02–3.34], *p*-value = 0.043 and aOR = 2.509, IC95% [1.41–2.29], *p*-value = 0.002, respectively). Other geriatric variables such as cognitive and mood impairment, TUG, OLBT or handgrip strength were associated with increased BCI levels (detailed in Table S3).

### 3.3. BCI and Survival

#### 3.3.1. Total Population

**Figure 2:**
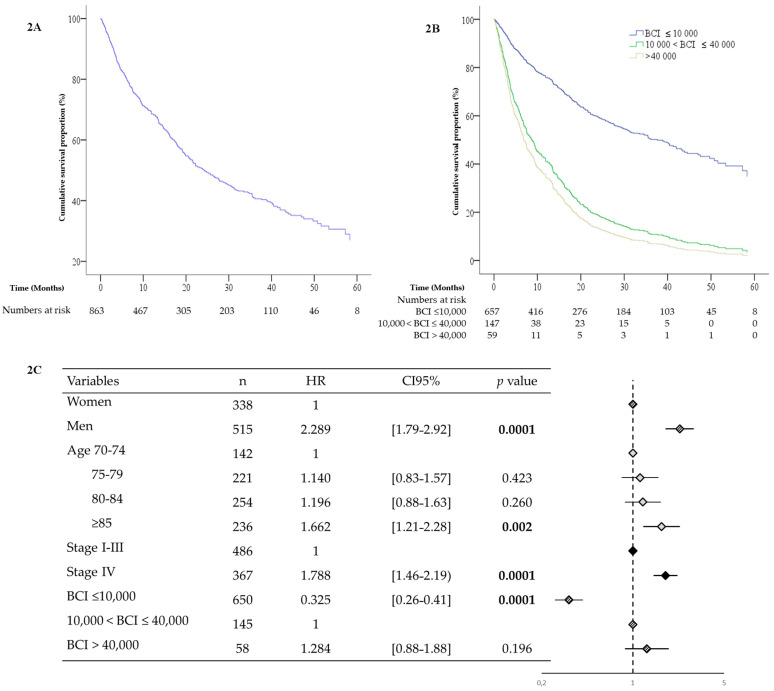
Overall survival time (panel **A**), overall survival time according to BCI level and BCI group (panel **B**), multivariate COX survival analysis (panel **C**). BCI: B12/CRP index; aHR: adjusted hazard ratio. Significant *p*-values were highlighted in bold.

Median survival time since date of CGA was 24.8 months ± 2.2 months (CI95% [20.6–29.1]) (Figure 2 panel 2A). Low BCI group median survival time was 35.5 ± 3.3 months (IC95% [29.0–41.9]); mid BCI median survival time was 6.2 ± 0.8 months (IC95% [4.7–7.7]) and high BCI median survival time was 5.3 ± 0.8 months (CI95% [3.8–6.8]). The log rank was 0.0001 (Figure 2 panel 2B). 

Median survival times in the mid- and high-BCI groups were very similar. In order to confirm this and to test whether there was any difference between the low- and mid-BCI groups, we performed a multivariable Cox model analysis using the mid-BCI group as a reference. The analysis confirmed the similar survival times (aHR = 1.305, CI95% [0.89–1.91], *p*-value = 0.167), and highlighted a significantly longer survival time in the low-BCI group (aHR = 0.327, CI95% [0.26–0.42], *p*-value = 0.0001) irrespective of gender, age, and cancer stage. Male gender and cancer stage also were predictive factors of mortality (aHR = 2.294, CI95% [1.80–2.92], *p*-value = 0.0001 and aHR = 1.775, CI95% [1.45–2.35], *p*-value = 0.0001 respectively) (Figure 2 panel 2C).

#### 3.3.2. Survival According to Cancer Site

We performed a univariate Kaplan Meier analysis (Figure S1) which provided similar results to those of the total population analysis. However, for several cancer sites the number of people affected was too small to be able to draw conclusions.

**Figure 3:**
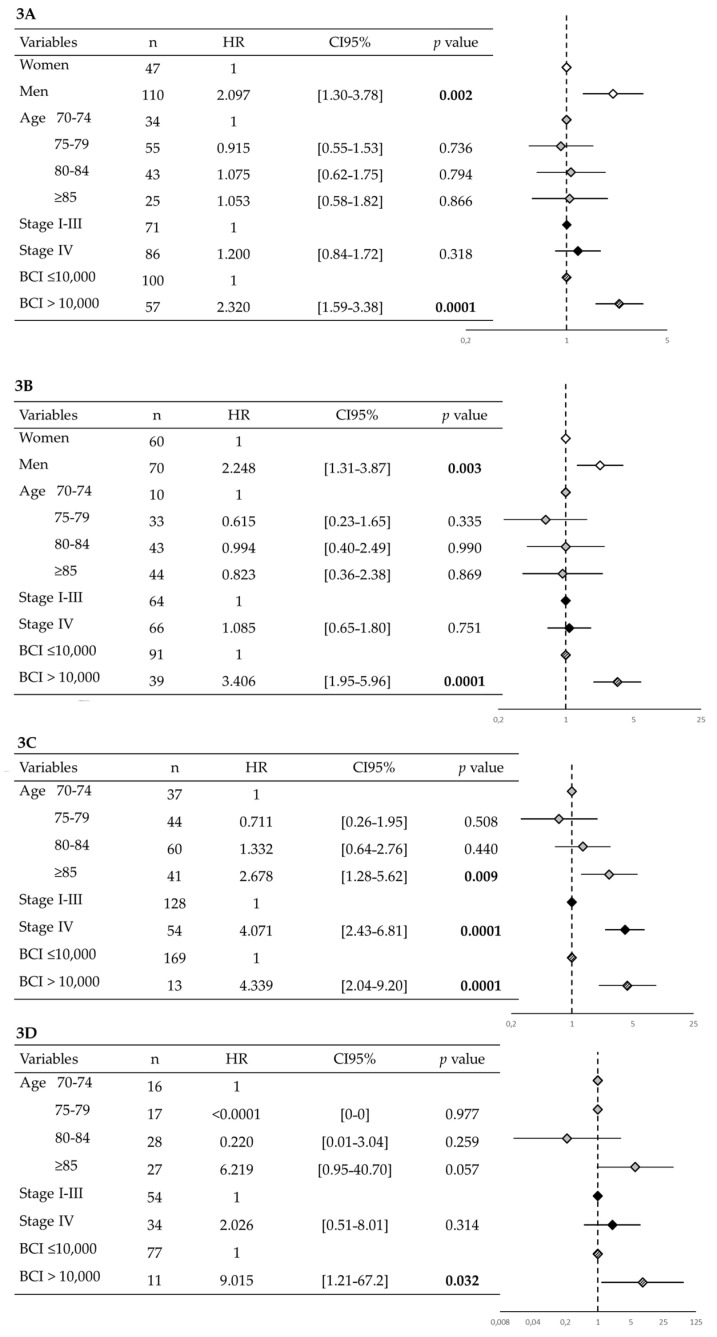
Multivariate COX Analysis in the four prominent sites of our population: thoracic (panel 3**A**), gastro-intestinal (panel 3**B**), prostate (panel 3**C**) and breast (panel 3**D**). Gender was excluded from the Cox analysis of prostate (only males) and breast (too few male to perform analysis *n* = 3). Significant *p*-values were highlighted in bold.

Given the low number of people in the high-BCI group (*n* = 59) and the lack of any significant difference between mid- and high-BCI groups (aHR = 1.305, CI95% [0.89–1.91], *p*-value = 0.167) in terms of survival time, we pooled both groups for the multivariable Cox model survival analysis stratified by cancer site. Only thoracic, gastrointestinal, prostate, and breast cancers had a sufficient number of patients to permit stratified multivariate analysis (Figure 3). A BCI > 10,000 was significantly associated with mortality risk in these four cancer types (thoracic: OR = 2.320, CI95% [1.59–3.38], *p*-value = 0.0001; gastrointestinal: aHR = 3.406, CI95% [1.95–5.96], *p*-value = 0.0001; prostate: aHR = 4.339, CI95% [2.04–9.20], *p*-value = 0.0001; breast: aHR = 9.015, CI95% [1.21–67.2], *p*-value = 0.032).

## 4. Discussion

In our cohort study sample of 863 older patients with cancer (all types, all stages), we observed a strong association between poor overall survival time and a high BCI value (BCI > 40,000) before systemic cancer treatment initiation. Median survival time was longer in the low-BCI (BCI ≤ 10,000) patient group than in the mid-BCI (10,000 < BCI ≤ 40,000) and high-BCI (>40,000) groups. Our results indicated that a BCI > 10,000 (pooling the mid- and high-BCI groups) was a predictive factor for overall survival in patients with prostate, breast, gastrointestinal and thoracic cancers. To our knowledge, this is the first study to show such an association in older people who have cancer. 

The prevalences of mid- and high-BCI values in our population of older cancer patients were lower than those previously described in populations with advanced cancer in the palliative care context (17% versus 21 to 33% (mid), and 6.9% versus 39 to 52% (high). 

We showed an association between high BCI values and geriatric impairment. Functional status, cognitive status, mood, mobility impairment, muscle strength impairment and malnutrition were all associated with high BCI levels. Moreover, functional status impairment (impaired ADL and/or IADL) and malnutrition were independently associated with mid- and high-BCI values. These associations have not been previously described. However, associations between impaired geriatric functions and B12 and CRP levels (considered separately) have already been shown in the literature; specifically a high CRP level has been found to be a good predictor of decreased physical and cognitive performance in the older population [29]. Furthermore, cognitive states in the older population may be affected by high concentrations of vitamin B12. More generally, functional status impairment and malnutrition are associated with poor outcomes in older patients treated with systemic treatment [30,31,32]. 

A high BCI value is a marker of deterioration of general condition in older adults. As BCI is directly proportional to CRP levels, its association with malnutrition could also be linked to inflammatory and/or cancer-induced malnutrition [33,34] and sarcopenia [27]. Our results showed an association between high BCI and handgrip strength impairment and gait speed which are also markers of sarcopenia [27]. The higher the BCI value, the more it is significantly associated with impairment in several frailty makers; accordingly, it could be an interesting biological tool to screen frailty in this population.

High BCI scores were previously associated with higher 90-day mortality risk in older patients with advanced or metastatic cancer [10,14,35]. High vitamin B12 level is also a strong predictor of mortality in older patients without cancer [11].The first ever study on BCI aimed to assess its usefulness as a prognostic index of three-month mortality in patients with advanced cancer [10]. In subsequent work, Kelly et al. (2007) and Tavares et al. (2010) also assessed this usefulness in terms of three-month mortality. Unlike our sample, theirs had younger patients (median age 68.7 years and 71 years, respectively) and were all in palliative care with a very short median survival time (45 and 44 days, respectively) [14,35]. To our knowledge, our study is the first to show the link between both mid- and high-BCI values and survival time in systematically-treatable older cancer patients. 

Unlike our results, previous studies did not find any association between mid BCI values and an increased risk of mortality in the older cancer population, nor did they find a difference between low or mid BCI values and survival time. We showed that survival in patients with a low BCI value was significantly longer than in mid-BCI patients. Furthermore, our study sample profile was different than those of previous study populations; specifically, our patients were, on average, 10 years older with advanced but treatable cancers. Moreover, all were enrolled before the initiation of systemic therapy, and 67.7% were proposed chemotherapy. Patients with a BCI ≤ 10,000 had a median survival time of more than two years. 

In stratified analysis, we verified that a BCI > 10,000 could be considered a threshold marker for poor survival prognosis for the four most common tumor sites in older populations [36]): thoracic, gastrointestinal, prostate and breast cancers. 

### 4.1. Perspectives

A BCI > 10,000 threshold seems to be a particularly promising biological prognostic factor of poor outcomes in older adults with cancer before systemic treatment initiation. Other studies are needed to study the prognostic value of BCI in the older in contexts other than cancer. 

### 4.2. Strength and Limitations

One strength of our study was that biological data were systematically collected at the same time as CGA before initiation of oncological systemic treatment. Furthermore, we had a relatively large sample (863 patients) of older patients with cancer, assessed in an outpatient setting. Measuring BCI is an easy and convenient procedure which can be routinely performed with CGA before oncological treatment in older patients.

This study has limitations. First, its monocentric design and the fact that all included patients were referred for a CGA (i.e., were suspected of frailty) prevented us from being able to verify the validity of our findings. Further analyzes on larger samples are needed to confirm our results, especially those related to specific cancer sites. Second, due to retrospective data collection, some clinical information was missing, such as metastases site, and we only collected data for proposed systemic treatment. Accordingly, the association between BCI level and the actual systemic treatment delivered was not evaluated. Finally, data on causes of death were not collected. Due to lack of details in the clinical data collected, heterogeneity of the patients and the small number of patients with high BCI, we chose to validate pre-existing BCI categories in our study population. Other BCI thresholds may be more accurate and new prospective studies are needed to refine our results and to determine optimal BCI thresholds for older patients with cancer.

## 5. Conclusions

A BCI > 10,000 is a useful biological prognostic factor for poor survival outcomes in older people with cancer before initiation of systemic treatment. In particular, a BCI > 10,000 seems to be a prognostic factor for survival in thoracic, gastrointestinal, prostate and breast malignancies. Higher BCI values were associated with greater geriatric frailty such as poorer functional status and malnutrition. The BCI could also be used as a marker of geriatric impairment in older cancer patients. Finally, BCI is a biological index which could help clinicians in oncological treatment decision-making for older patients with cancer, and should be integrated into the Comprehensive Geriatric Assessment.

## Figures and Tables

**Table 1:** Descriptive and comparative analysis.

Variables	Total Population (*n* = 863)		BCI ≤ 10,000 (*n* = 657)		10,000 < BCI ≤ 40,000 (*n* = 147)		BCI > 40,000 (*n* = 59)		*p*-Value
	*n*	%	*n*	%	*n*	%	*n*	%	
Gender									
Women	341	39.5	269	40.9	49	33.3	23	39.0	0.232
Men	522	60.5	388	45.0	98	66.7	36	61.0	
Age (years)									
70–74	142	16.5	114	17.4	17	11.6	11	18.6	0.152
75–79	223	25.8	162	24.7	46	31.3	15	25.4	
80–84	257	29.8	190	28.9	44	29.9	23	39.0	
≥85	241	27.9	191	29.1	40	27.2	10	16.9	
Stage IV cancer (*n* = 853)	367	42.5	245	37.7	86	59.3	36	62.1	**0.0001**
Cancer type (*n* = 862)									
Prostate	184	21.3	171	26.0	11	7.5	2	3.4	**0.0001**
Thoracic	183	21.2	112	17.0	52	35.4	19	32.2	
Gastrointestinal	131	15.2	92	14.0	25	17.1	14	23.7	
Breast	90	10.4	78	11.9	8	5.4	4	6.8	
Head and neck	67	7.8	45	6.8	20	13.7	2	3.4	
Female reproductive organs	56	6.5	43	6.5	6	4.1	7	11.9	
Urological	50	5.8	38	5.8	8	5.5	4	6.8	
Hematological	51	5.9	37	5.6	10	6.8	4	6.8	
Skin	35	4.1	31	4.7	4	2.7	3	5.1	
Other	15	1.7	10	1.5	2	1.4	3	5.1	
Autonomy (*n* = 860)									
Unimpaired ADL-IADL	323	37.6	284	43.4	33	22.4	6	10.2	**0.0001**
Impaired ADL or IADL	224	26.0	174	26.6	39	26.5	11	18.6	
Impaired ADL and IADL	313	36.4	196	30.0	75	51.0	42	71.2	
Cognitive impairment (*n* = 843)	538	63.8	389	60.3	106	73.6	43	79.6	**0.0001**
Mood impairment (*n* = 845)	391	46.3	270	41.7	89	60.8	34	61.8	**0.0001**
Handgrip Strength ^1^ (*n* = 841)	371	44.1	242	37.5	89	63.6	40	71.4	**0.0001**
Mobility impairment (*n* = 862)									
TUG (>20 s) (*n* = 824)	345	41.9	234	37.3	72	51.4	39	69.6	**0.0001**
OLBT (<5 s) (*n* = 766)	504	65.8	373	63.1	86	69.4	45	88.2	**0.001**
Gait speed (<0.8 m/s) (*n* = 762)	390	51.2	299	49.9	58	49.6	33	71.7	**0.016**
Falls ^2^ (*n* = 862)	155	18.0	108	16.4	31	21.1	16	27.6	0.059
Malnutrition ^3^ (*n* = 863)	290	33.6	155	23.6	87	59.2	48	81.4	**0.0001**
MNA (<17/30)	119	14.4	62	9.8	40	28.8	17	30.9	**0.0001**
BMI (<21) (*n* = 860)	165	19.1	100	15.2	46	31.5	19	32.8	**0.0001**
Albumin (<35 g/L) (*n* = 855)	129	15.1	42	6.4	46	31.7	41	70.7	**0.0001**
Polypharmacy ^4^ (*n* = 862)	557	64.6	399	60.8	114	77.6	44	74.6	**0.0001**
Severe comorbidities ^5^ (*n* = 862)	466	39.9	344	52.4	90	61.2	32	54.2	0.156
Anemia ^6^ (*n* = 860)	546	63.5	380	58.1	114	77.6	52	63.5	**0.0001**
Thrombocytopenia ^7^ (*n* = 858)	86	10.0	69	10.6	10	6.8	7	10.0	0.343
B12 ^8^									
Normal	697	80.8	552	84.0	113	76.9	32	54.2	**0.0001**
Hypo	38	5.8	38	5.8	-	-	-	-	
Hyper	128	14.8	67	10.2	34	23.1	27	45.8	
CRP (>5 mg/L)	464	53.8	258	39.3	147	100	59	100	**0.0001**
Lymphopenia ^9^ (*n* = 853)	337	39.0	238	36.5	66	46.5	33	55.9	**0.002**
Severe renal deficiency ^10^ (*n* = 863)	107	12.4	66	10.0	31	21.1	10	16.9	**0.001**

BCI: B12/CRP index; ADL: Activity of daily living; IADL: Instrumental activity of daily living; TUG: Timed ‘Up and Go’ test; OLBT: One leg balance test; BMI: body mass index. ^1^ Impaired Handgrip Strength was defined as 27 kg/men, 16 kg/women. ^2^ Fallwas defined as one or more falls within the three months prior to CGA. ^3^ Malnutrition is defined as MNA < 17/30, and/or BMI < 21 and/or albumin <35 g/L. ^4^ Polypharmacy is defined as five or more drugs. ^5^ Severe comorbidities are define as one or more comorbidities rating 3 or more on the CIRS. ^6^ Anemia was defined as hemoglobin levels <13 g/dL for men and <12 g/dL for women. ^7^ Thrombocytopenia was defined as platelets levels <150 Giga/L. ^8^ B12 vitamin normal levels are between 145 and 569 pmol/L (HypoB12 < 145 pmol/L and HyperB12 > 569 pmol/L). ^9^ Lymphopenia was defined as lymphocytes levels <1.26 Giga/L. ^10^ Severe renal deficiency was defined as Cockcroft glomerular filtration rate <30 mL/min. Significant *p*-values were highlighted in bold.

**Table 2:** Association between BCI level and geriatric characteristics (autonomy, nutrition, gait speed): multinomial logistic regression (Reference group is BCI < 10,000).

Variables	10,000 < BCI ≤ 40,000 (*n* = 147)			BCI > 40,000 (*n* = 59)		
	aOR	CI95%	*p*-Value	aOR	CI95%	*p*-Value
**MODEL A (*n* = 853)**						
Gender						
Women	1			1		
Men	1.950	[1.30–2.93]	**0.001**	1.843	[1.02–3.34]	**0.043**
Age (years)						
70–74	1			1		
75–79	1.901	[1.01–3.56]	**0.045**	0.973	[0.41–2.29]	0.973
80–84	1.324	[0.71–2.49]	0.382	0.963	[0.43–2.15]	0.927
≥85	1.051	[0.55–2.00]	0.881	0.310	[0.12–0.79]	**0.014**
Stage						
I–III	1			1		
IV	2.452	[1.68–3.59]	**0.0001**	2.509	[1.41–2.29]	**0.002**
Autonomy						
Unimpaired ADL-IADL	1			1		
Impaired ADL or IADL	2.509	[1.41–4.48]	**0.005**	3.343	[1.20–9.29]	**0.021**
Impaired ADL and IADL	4.069	[2.50–6.63]	**0.0001**	14.375	[5.80–35.66]	**0.0001**
**MODEL B (*n* = 853)**						
Gender						
Women	1			1		
Men	1.9000	[1.26–2.87]	**0.002**	1.659	[0.92–3.00]	0.094
Age (years)						
70–74	1			1		
75–79	2.027	[1.06–3.86]	**0.032**	1.094	[0.46–2.63]	**0.840**
80–84	1.492	[0.79–2.83]	0.222	1.196	[0.53–2.71]	0.668
≥85	1.455	[0.76–2.79]	0.258	0.550	[0.21–1.41]	0.212
Stage						
I–III	1			1		
IV	2.226	[1.51–3.29]	**0.0001**	2.275	[1.26–4.10]	**0.006**
Malnutrition						
No	1			1		
Yes	4.969	[3.3–7.37]	**0.0001**	14.348	[7.16–28.74]	**0.0001**
**MODEL C (*n* = 853)**						
Gender						
Women	1			1		
Men	1.892	[1.21–2.97]	**0.006**	1.914	[0.97–3.76]	0.060
Age (years)						
70–74	1			1		
75–79	1.476	[0.77–2.84]	0.244	0.779	[0.32–1.92]	0.588
80–84	1.359	[0.71–2.59]	0.351	0.915	[0.40–2.12]	0.915
≥85	1.266	[0.65–2.48]	0.492	0.487	[0.18–1.29]	0.149
Stage						
I–III	1			1		
IV	2.738	[1.81–4.14]	**0.0001**	2.369	[1.26–4.44]	**0.007**
Gait speed (<0.8 m/s)						
No	1			1		
Yes	1.027	[0.68–1.56]	0.889	2.762	[1.40–5.45]	**0.003**

BCI: B12/CRP index; aOR: adjusted odd ratio; malnutrition: BMI < 21 and/or albumin levels < 35 g/L and/or MNA < 17. Significant *p*-values were highlighted in bold

## Data Availability

The data presented in this study are available on request from the corresponding author. The data are not publicly available due to privacy restrictions.

## Supplementary Materials

The following supporting information can be downloaded at https://www.mdpi.com/article/10.3390/cancers14010169/s1, Table S1: Comparison of older patients included in the analysis and those excluded for lack of B12 or CRP dosage or lost to follow-up, Table S2: Oncological treatment proposal for the 863 patients, Table S3: Association between BCI level and other geriatric characteristics (social isolation, polypharmacy, cognitive disorders, mood impairment, TUG, OLBT, handgrip strength): multinomial logistic regression, Figure S1: Kaplan Meier analysis, overall survival proportion according to BCI groups for each cancer localization.
