# Supplementary material for: Prognostic Value of the B12/CRP Index in Older Systemically Treatable Cancer Patients
Source: Cancers (Basel). 2021 Dec 30;14(1):169. doi: 10.3390/cancers14010169 (PMC8750046; doi:10.3390/cancers14010169)
Supplement: Supplementary file 1 [file cancers-14-00169-s001.zip › cancers-1499998-supplementary.pdf]

cancers MDPI

Supplementary Materials

Prognostic Value of the B12/CRP Index in Older Systemically Treatable Cancer Patients

Coline Montegut, Florian Correard, Emilie Nouguerède, Dominique Rey, Thomas Chevalier, Marie Meurer, Jean-Laurent Deville, Marjorie Baciuchka, Vincent Pradel, Laurent Greillier, Patrick Villani and Anne-Laure Couderc

Table S1. : Comparison of older patients included in the analysis and those excluded for lack of B12 or CRP dosage or lost to follow-up.

	**Included (*n* = 863)**		**Excluded (*n* = 311)**		***p*-value**
	n	**%**	***n***	**%**	
**Gender** Women	341	39.5	117	37.6	0.557
Men	522	60.5	194	62.4	
**Age (years)** 70-74	142	16.5	81	26.0	**0.001**
75-79	223	25.8	85	27.3	
80-84	257	29.8	81	26.0	
≥85	241	27.9	64	20.6	
**Stage IV cancer** (*n* = 1158)	486	57.0	149	51.1	**0.014**
**Cancer type** (*n* = 1174)					
Prostate	184	21.3	42	13.5	**0.0001**
Thoracic	183	21.2	92	29.6	
Gastrointestinal	131	15.2	42	13.5	
Breast	90	10.4	18	5.8	
Head and neck	67	7.8	34	10.9	
Female reproductive organs	56	6.5	15	4.8	
Urological	50	5.8	25	8.0	
Hematological	51	5.9	11	3.5	
Skin	36	4.2	27	8.7	
Other	15	1.7	5	1.6	
**Autonomy** (*n* = 1174)					
Unimpaired ADL-IADL	323	37.4	120	38.6	0.801
Impaired ADL or IADL	224	26.0	74	23.8	
Impaired ADL and IADL	313	36.3	115	37.0	
**Cognitive impairment** (*n* = 1137)	538	63.8	187	63.6	0.948
**Mood impairment** (*n* = 1149)	391	46.3	147	48.4	0.533
**Handgrip Strength**[1] (*n* = 1137)	470	55.9	176	59.5	0.286
**Mobility impairment**					
TUG *(>20s)* (*n* = 824)	345	41.9	152	50.8	**0.007**
OLBT *(<5s)* (*n* = 766)	504	65.8	242	86.4	**0.0001**
Gait speed *(<0.8m/s)* (*n* = 762)	390	51.2	144	55.4	0.241
Falls[2] (*n* = 862)	155	18.0	65	21.0	0.248
**Malnutrition**[2] (*n* = 863)	290	33.6	126	40.6	**0.026**
**Polypharmacy**[3] (*n* = 862)	557	64.6	201	64.8	0.944
**Severe comorbidities**[4] (*n* = 862)	466	54.1	111	35.7	**0.0001**

Table S2. Oncological treatment proposal for the 863 patients.

Treatments	*n*	%	Treatments	*n*	%
**Chemotherapy (CT)**	584	67.7	**Hormonotherapy (HT)**	210	24.3
Alone	427	49.5	Alone	47	5.4
Radio-chemo	115	13.3	Radio-HT	139	16.1
Radio-Chemo and hormonotherapy	14	1.6	HT and radio-chemo	14	1.6
CT and/or hormonotherapy (HT)	13	1.5	HT and/or CT	13	1.5
CT and/or targeted therapy (TT)	10	1.2	HT and/or TT	10	1.2
CT and/or Immunotherapy (IT)	5	1.5	**Targeted therapy (TT)**	51	5.9
**Immunotherapy (IT)**	41	4.8			
Alone	33	3.8	Alone	38	4.4
IT and/or CT	5	1.5	Radio-TT	3	0.3
IT and/or TT	4	0.5	TT and/or CT	5	1.5
Radio-IT	4	0.5	TT and/or HT	6	0.7
**Others**	5	0.6	TT and/or IT	4	0.5

Table S3. Association between BCI level and other geriatric characteristics (social isolation, polypharmacy, cognitive disorders, mood impairment, TUG, OLBT, handgrip strength): multinomial logistic regression (Reference group is BCI < 10 000).

		**10 000< BCI ≤40 000 (*n* = 147)**			**BCI > 40 000 (*n* = 59)**		
MODEL D (n = 853)		**aOR**	**CI95%**	***p*-value**	**aOR**	**CI95%**	***p*-value**
**Gender**	Women	1			1		
	Men	1.542	[1.05-2.27]	**0.029**	1.179	[0.67-2.06]	0.565
**Age (years)**	70-74	1			1		
	75-79	1.513	[0.81-2.81]	0.192	0.583	[0.24-1.43]	0.237
	80-84	1.485	[0.80-2.75]	0.209	1.184	[0.55-2.55]	0.667
	≥85	1.844	[1.00-3.42]	0.052	0.952	[0.42-2.17]	0.952
**Stage**	I-III	1			1		
	IV	2.522	[1.74-366]	**0.0001**	2.696	[0.15-4.71]	**0.001**
**Social isolation**	Absent	1			1		
	Present	1.471	[0.92-2.36]	0.109	1.370	[0.68-2.76]	0.379
MODEL E (n = 852)		aOR	CI95%	*p*-value	aOR	CI95%	*p*-value
**Gender**	Women	1			1		
	Men	1.458	[0.99-2.16]	0.058	1.117	[0.64-1.96]	0.700
**Age (years)**	70-74	1			1		
	75-79	1.415	[0.76-2.65]	0.278	0.549	[0.22-1.35]	0.190
	80-84	1.426	[0.78-2.65]	0.262	1.144	[0.53-2.47]	0.733
	≥85	1.843	[0.99-3.42]	0.053	0.947	[0.42-2.16]	0.896
**Stage**	I-III	1			1		
	IV	2.415	[1.66-3.51]	**0.0001**	2.609	[1.50-4.57]	**0.001**
**Polypharmacy[1]**	< 5 drugs	1			1		
	≥ 5 drugs	2.021	[1.32-3.10]	**0.01**	1.777	[0.86-3.30]	0.068
MODEL F (n = 833)		aOR	CI95%	*p*-value	aOR	CI95%	*p*-value
**Gender**	Women	1			1		
	Men	1.576	[1.06-2.34]	**0.024**	1.255	[0.70-2.26]	0.450
**Age (years)**	70-74	1			1		
	75-79	1.182	[0.62-2.24]	0.607	0.401	[0.15-1.07]	0.067
	80-84	1.381	[0.74-2.58]	0.310	1.107	[0.49-2.48]	0.805
	≥85	1.844	[0.99-3.43]	0.054	0.965	[0.41-2.28]	0.936
**Stage**	I-III	1			1		
	IV	2.713	[1.85-3.97]	**0.0001**	2.779	[1.54-5.01]	**0.001**
**Cognitive disorders**	Absent	1			1		
	Present	2.034	[1.33-3.10]	**0.01**	2.990	[1.49-6.01]	**0.002**
MODEL G (n = 835)		aOR	CI95%	*p*-value	aOR	CI95%	*p*-value
**Gender**	Women	1			1		
	Men	1.648	[1.11-2.16]	**0.014**	1.360	[0.76-2.45]	0.305

**Age (years)**	70-74	1			1		
	75-79	1.173	[0.78-2.77]	0.230	0.734	[0.29-1.88]	0.519
	80-84	1.458	[0.78-2.72]	0.237	1.326	[0.58-3.05]	0.507
	≥85	1.874	[1.00-3.50]	0.049	1.209	[0.51-2.89]	0.670
**Stage**	I-III	1			1		
	IV	2.416	[1.65-3.54]	**0.0001**	2.476	[1.39-4.41]	**0.002**
**Mood impairment**	Absent	1			1		
	Present	2.201	[1.50-3.23]	**0.0001**	2.171	[1.21-3.88]	**0.009**
MODEL H (n = 816)		aOR	CI95%	*p*-value	aOR	CI95%	*p*-value
**Gender**	Women	1			1		
	Men	1.628	[1.09-2.42]	**0.017**	1.274	[0.71-2.28]	0.414
**Age (years)**	70-74	1			1		
	75-79	1.148	[0.60-2.19]	0.675	0.398	[0.16-2.32]	0.057
	80-84	1.317	[0.70-2.46]	0.388	1.026	[0.45-2.32]	0.951
	≥85	1.711	[0.92-3.20]	0.092	0.851	[0.36-2.04]	0.717
**Stage**	I-III	1			1		
	IV	2.210	[1.51-3.24]	**0.0001**	2.051	[1.15-3.66]	**0.015**
**TUG**	≤20s	1			1		
	>20s	1.834	[1.24-2.72]	**0.002**	4.345	[2.34-8.06]	**0.0001**
MODEL I (n = 758)		aOR	CI95%	*p*-value	aOR	CI95%	*p*-value
**Gender**	Women	1			1		
	Men	1.864	[1.21-2.87]	**0.005**	1.605	[0.87-2.98]	0.134
**Age (years)**	70-74	1			1		
	75-79	1.196	[0.60-2.40]	0.615	0.362	[0.14-0.96]	**0.040**
	80-84	1.351	[0.70-2.63]	0.375	0.754	[0.32-1.75]	0.511
	≥85	1.732	[0.90-3.35]	0.103	0.803	[0.33-1.95]	0.629
**Stage**	I-III	1			1		
	IV	2.429	[1.62-3.65]	**0.0001**	2.344	[1.27-4.31]	**0.006**
**OLBT**	≥ 5 sec	1			1		
	<5 sec	1.297	[0.82-2.03]	0.257	5.026	[2.05-12.33]	**0.0001**
MODEL J (n = 831)		aOR	CI95%	*p*-value	aOR	CI95%	*p*-value
**Gender**	Women	1			1		
	Men	1.459	[0.97-2.19]	0.068	1.304	[0.72-2.36]	0.382
**Age (years)**	70-74	1			1		
	75-79	0.792	[0.40-1.55]	0.494	0.256	[0.09-0.70]	0.008
	80-84	1.069	[0.56-2.03]	0.839	0.861	[0.38-1.97]	0.723
	≥85	1.604	[0.85-3.03]	0.146	0.912	[0.38-2.17]	0.835
**Stage**	I-III	1			1		
	IV	2.638	[1.78-3.90]	**0.0001**	2.868	[1.59-5.17]	**0.0001**
**Handgrip strength[2]**	Normal	1			1		
	Impaired	3.414	[2.26-5.17]	**0.0001**	5.261	[2.79-9.94]	**0.0001**

BCI: B12/CRP index; TUG: Timed 'Up and Go' test; OLBT: One leg balance test;.[1] Polypharmacy define as 5 or more drugs.
[2] Impaired Handgrip Strength was defined as 27kg / men, 16kg / women.

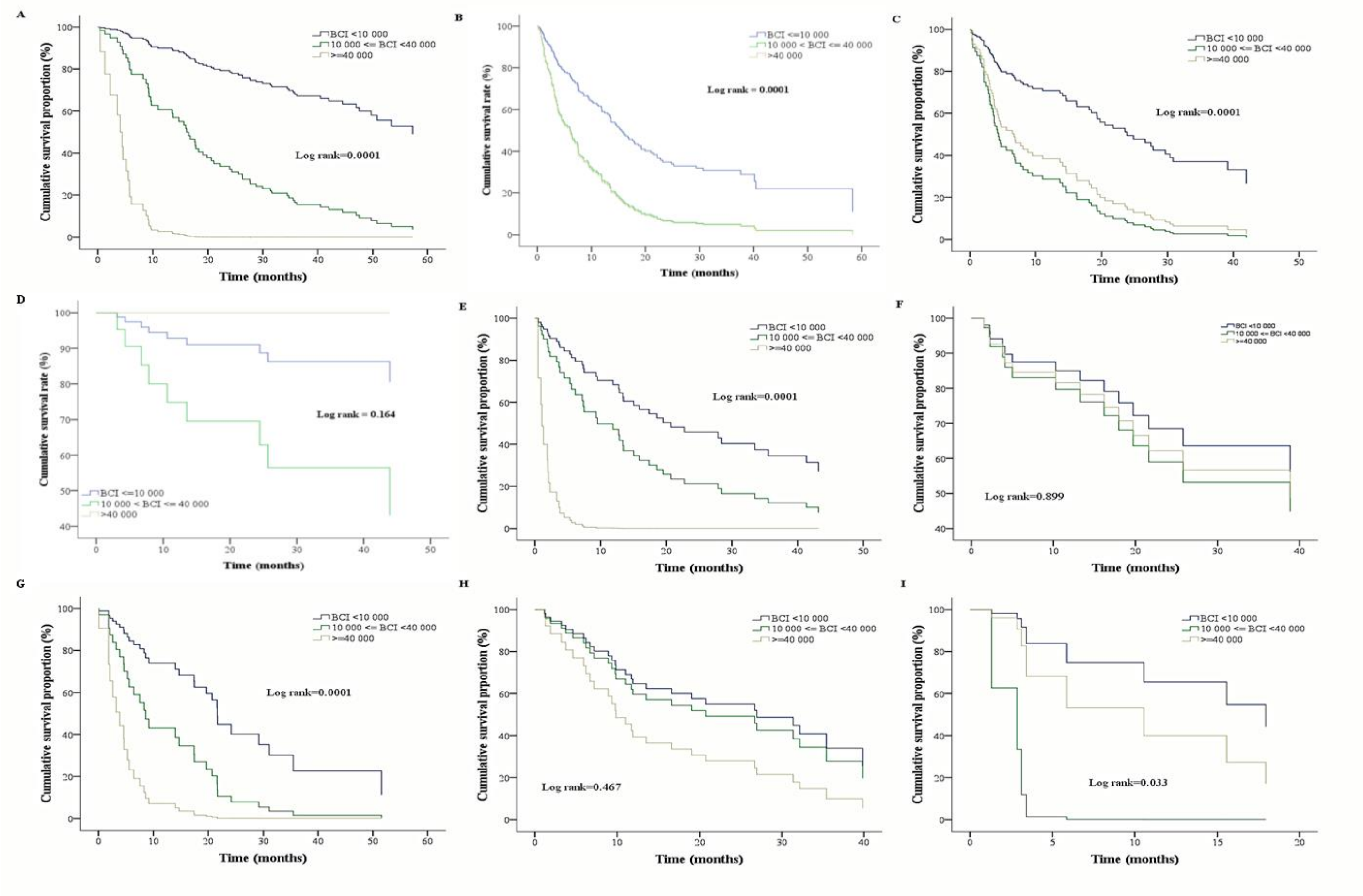

Figure S1. Kaplan Meier analysis, overall survival proportion according to BCI groups for each cancer localization (panel **A**: prostate; **B**: thoracic; **C**: gastro-intestinal; **D**: breast; **E**: head and neck; **F**: female reproductive organs; **G**: urology (bladder and kidney); **H**: hematological malignancies; **I**: skin).
